# Attitudes of Canadian Colorectal Cancer Care Providers towards Liver Transplantation for Colorectal Liver Metastases: A National Survey

**DOI:** 10.3390/curroncol29020054

**Published:** 2022-01-28

**Authors:** Keegan Guidolin, Woo Jin Choi, Filomena Servidio-Italiano, Fayez Quereshy, Gonzalo Sapisochin

**Affiliations:** 1 Department of Surgery, University of Toronto, Toronto, ON M5G 2C4, Canada; keegan.guidolin@mail.utoronto.ca (K.G.); woojin.choi@mail.utoronto.ca (W.J.C.); fayez.quereshy@uhn.ca (F.Q.); 2 Department of Surgery, University Health Network, Toronto, ON M5G 1L7, Canada; 3 Institute of Biomedical Engineering, University of Toronto, Toronto, ON M5S 3G9, Canada; 4 Institute for Health Policy, Management, and Evaluation, University of Toronto, Toronto, ON M5T 3M6, Canada; 5 Multi-Organ Transplant and HPB Surgical Oncology, Division of General Surgery, University Health Network, Toronto, ON M5G 1L7, Canada; 6 Colorectal Cancer Resource and Action Network, 700-2 Bloor St. W., Toronto, ON M4W 3E2, Canada; filomena.s@ccran.org

**Keywords:** living donor liver transplant, colorectal liver metastasis, survey, awareness, attitudes

## Abstract

Up to 50% of colorectal cancer (CRC) patients develop colorectal liver metastases (CRLM). The aim of this study was to gauge the awareness and perception of liver transplantation (LT) for non-resectable CRLM, and to describe the current referral patterns and management strategies for CRLM in Canada. Surgeons who provide care for patients with CRC were invited to an online survey through the Canadian Association of General Surgeons, the Canadian Society of Colon and Rectal Surgeons, and the Canadian Society of Surgical Oncology. Thirty-seven surveys were included. The most utilized management strategy for CRLM was to refer to a hepatobiliary surgeon for assessment of metastectomy (78%), and/or refer to medical oncologists for consideration of chemotherapy (73%). Among the respondents, 84% reported that their level of knowledge about LT for CRLM was low, yet the perception of exploring the option of LT for non-resectable CRLM seemed generally favorable (81%). The decision to refer for consideration of LT for CRLM treatment seemed to depend on patient-specific factors and the local hepatobiliary surgeon’s recommendation. Providing CRC care providers with educational materials on up-to-date CRLM management may help raise the awareness of the use of LT for non-resectable CRLM.

## 1. Introduction

Colorectal cancer (CRC) is the third most common cause of cancer death worldwide, with over 2 million new cases diagnosed each year, and more than 1 million related deaths [1]. The liver is the most common site of metastasis for CRC, and it is reported that up to 50% of CRC patients develop colorectal liver metastases (CRLM) [2,3]. The combination of surgery and chemotherapy is the only accepted curative treatment option; however, it has been estimated that 60–80% of patients with CRLM are not candidates for liver resection for reasons such as insufficient liver remnant volume or high tumor burden [4]. In cases where the colorectal metastases are isolated to the liver, the total hepatectomy resulting from liver transplantation (LT) offers a chance of cure by removing all disease [5]. Currently, CRLM is considered an absolute contraindication for LT at most centers, leaving palliative treatment as the only remaining option [5].

Oslo University Hospital Group was the first to show promising results following LT for CRLM, with a 60% 5-year OS rate in the Secondary Cancer (SECA) I trial [6]. The SECA-I trial’s main inclusion criteria consisted of “completed radical excision of the primary tumor, good performance status (ECOG score 0 or 1), and minimum 6 weeks of chemotherapy”, and the exclusion criteria consisted of “weight loss of more than 10% and other malignancies” [6]. SECA-I data was then used to show a similar 5-year OS rate (75% vs. 76%) between the “low-risk” liver-only CRLM group receiving LT, and patients receiving LT for hepatocellular carcinoma within the Milan Criteria [7]. The “low-risk” group consisted of patients who met three or less of: (1) CEA level above 80 μg/L, (2) largest lesion greater than 5.5 cm, (3) less than 2 years between primary surgery and liver transplantation, and (4) progressive disease while receiving chemotherapy at time of transplantation [7]. Improved perioperative management of LT recipients, better understanding of immunosuppression physiology, utilization of living donor liver transplantations (LDLT), and evolving multidisciplinary care have since contributed to the marked improvement in post-LT outcomes [8]. The largest systematic review to date, consisting of 110 patients from 18 studies, reports up to 50.5% pooled 5-year overall survival (OS) for patients undergoing LT for CRLM [9]. Although the overall recurrence rate after LT for CRLM remains high (69.5%), the 5-year OS rate is also high, and will likely improve with better patient selection with respect to tumor biology to minimize disease recurrence [9]. 

LT for CRLM remains investigational. Our group at University Health Network (UHN), a tertiary referral center for CRLM, is conducting a prospective study using neoadjuvant chemotherapy and living donor liver transplantation (LDLT) for non-resectable CRLM from CRC (“LDLT CRLM trial,” NCT02864485) [10]. LDLT provides the advantage of offering a graft without affecting the existing deceased-donor organ pool, and facilitates logistical coordination between the operation and chemotherapy [11]. Given the novelty of this initiative, we sought to understand and measure cancer care providers’ level of awareness and perception of LT as a treatment option for patients with non-resectable CRLM in Canada. This question has critical importance, as disseminating innovations in healthcare is most heavily influenced by provider perceptions of the innovation and the perceived benefit of the change by the providers [12]. Their attitudes and opinion towards this prospective CRLM management strategy will be imperative to transition potentially eligible patients to this new management strategy, especially given the ethical considerations related to the use of scarce resources (i.e., organs) for investigational purposes such as CRLM while organ shortages persist across North America [13]. This would also provide an opportunity to understand the current referral and management preferences for patients with CRLM in Canada.

The aim of this study is to gauge the awareness and perception of LDLT for non-resectable CRLM in Canada, and to describe the current referral patterns and management strategies for CRLM in Canada.

## 2. Materials and Methods

### 2.1. Study Design and Questionnaire Development

A prospective, national study was conducted using an online survey to collect data. The questionnaire was developed after the three main domains of interest were identified (demographic information, practice patterns, and opinions). We underwent an iterative process of item generation based upon a multidisciplinary focus group session with the senior authors (GS and FQ) and CRC patient-focused organization president (FS) to ideate relevant questions within these domains. This was followed by a process of item reduction to minimize the number and length of these questions while distilling the essence of the items. We pilot tested this survey with three local colorectal surgeons and surgical oncologists, and incorporated their feedback into the design of the questionnaire, including their comments on the clarity and clinical sensibility of the questions. The final survey consisted of a total of 12 multiple-choice questions with 2 free-text comment boxes to provide elaboration and opinion (Appendix A).

### 2.2. Study Population and Questionnaire Distribution

This study sought to prospectively survey surgeons who provide care for patients with CRC; as such, we sent surveys to the general membership of two relevant Canadian surgical societies: the Canadian Association of General Surgeons (CAGS), and the Canadian Society of Colon and Rectal Surgeons (CSCRS), as part of their regular, online, mass newsletters. In addition, a standalone email was sent to the 200 members of the Canadian Society of Surgical Oncology (CSSO). CSSO sent the invitation to participate in the study to its membership by email, followed by reminder emails sent two and six weeks after the initial invitation email. Messaging within these all invitations asked that only surgeons who are providing care for patients with CRC should participate. The survey was available to complete online for eight weeks after initial invitations were sent out. This study protocol and survey were reviewed and approved by the University Health Network Research Ethics Board.

### 2.3. Data Collection and Analysis

All data were collected on a secure, web-based Research Electronic Data Capture (REDCap) data management platform [14]. The survey was completely anonymous, and voluntary consent was obtained prior to participation. All the respondent answers were self-reported online, including the free-text comments of opinions. Survey responses were analyzed on a question-by-question basis, with missing data reported as such. The included responses were pooled on the question-level. Count data were summarized as a proportion. Thematic extraction was conducted (KG, WC) to identify unique concepts and overarching themes for collected the opinions. All analyses were performed using Microsoft Excel (version 16.54, 2021).

## 3. Results

### 3.1. Demographic Characteristics

In total, 38 surgeons responded to our survey. One survey was excluded because the respondent did not meet the inclusion criteria of providing CRC care. Thirty-seven surveys were included in the analysis. Almost all (84%) respondents were between the ages of 31 and 50. The majority were male (59.5%), practiced in an academic setting (75.7%), and were trained as surgical oncologists (91.9%). Respondents were roughly evenly distributed in terms of the number of years in practice. For most respondents (83.8%), between 0 and 25% of their practice consisted of patients with CRLM. Demographic results are tabulated in Table 1.

### 3.2. Current Management Strategies for CRLM

The most commonly used strategy to manage patients with CRLM was referral to a hepatobiliary surgeon for assessment for metastectomy (78.4%), followed by referral to medical oncology for consideration for chemotherapy (73%). Most respondents (67.6%) also stated that they would present cases of CRLM at multidisciplinary tumor rounds. Three respondents (8.1%) included referral for other interventional therapy, such as portal vein embolization, tumor ablation, or hepatic artery infusion pump therapy, as potential management strategies. One respondent stated that they would refer the patient to a tertiary care surgical oncologist or colorectal surgeon, and one respondent included a referral to palliative care in their management plan. In addition, one respondent who also performs hepatobiliary surgery stated that they would manage the disease themselves, and two respondents stated that their management would be highly dependent upon the pattern of disease. Management strategies are tabulated in Table 2.

### 3.3. Knowledge, Opinions, and Attitudes towards LDLT for CRLM

Most respondents (83.8%) reported little to moderate knowledge about LDLT for CRLM; despite this, nearly half (45.9%) of respondents stated that they were aware of the indications for LDLT for CRLM. Over half (56.8%) of respondents perceived LDLT as an appropriate or absolutely appropriate management strategy for CRLM, whereas 27% perceived it as inappropriate or absolutely inappropriate. Surgeons were generally open to referring patients with CRLM for consideration of LDLT, with 48.7% stating that they might or might not consider referring, and 45.9% stating that they would definitely consider referring; 5.4% stated they would not consider referring patients for LDLT. A summary of the knowledge, opinions, and attitudes of surgeons towards LDLT for CRLM is tabulated in Table 3.

Thematic extraction of the opinions on the appropriateness of LDLT for CRLM (Table 4) revealed that a demand for a greater quantity or quality of data was common to all perceptions of appropriateness, with those who consider the therapy absolutely inappropriate, inappropriate, or neutral citing a perception that there is insufficient evidence or negative evidence for LDLT. These surgeons also cited the existence of better alternative therapies as a reason for considering LDLT to be inappropriate. Surgeons who considered LDLT to be absolutely inappropriate or inappropriate also cited concerns over the biology of the disease that would be implied by the presence of unresectable CRLM, and concerns about the risk–benefit profile of the intervention. Surgeons who considered LDLT to be appropriate or absolutely appropriate stated that LDLT may only be appropriate in the context of a clinical trial, that patients must be highly selected, and that long-term outcomes are still uncertain.

Thematic extraction of the opinions on surgeons’ openness to referral for LDLT for CRLM revealed significant overlap in opinion across categories (Table 5). Those who would not consider referral cited the existence of better alternative therapies, and the desire for more data. Those who might or might not consider referral also expressed a desire for more data. Both those who might or might not consider referral, and those who would definitely consider referral, expressed that their referral would depend on patient factors and on the opinion of their institutional hepatobiliary surgeon, and that they would only consider referral in the context of a clinical trial.

## 4. Discussion

In this national survey of surgeons who provide care for patients with CRC in Canada, we found that the most used management strategy for CRLM currently was to refer to an HPB surgeon for assessment for metastectomy and/or refer to medical oncologists for consideration for chemotherapy. The reported level of knowledge about LDLT for CRLM was generally low, yet the perception of exploring the option of LDLT for non-resectable CRLM seemed generally favorable. There was a clear demand for more level I clinical trial evidence for sufficient measurement of the risks and long-term outcomes of LDLT for CRLM, and for the comparison of LDLT to alternative therapy options. The referral decision for consideration of LDLT for CRLM treatment seems to depend largely on patient-specific factors and the local HPB surgeon’s recommendation.

To our knowledge, this is the first study exploring Canadian surgeons’ awareness and perception of LDLT for non-resectable CRLM. Results from this study can be used to understand how Canadian CRC care providers might plan to manage the projected surge of patients with CRLM due to: (1) the COVID-19 pandemic interrupting CRC screenings, and (2) the rising incidence of CRC in younger adults in Canada [15,16,17]. Our study participants’ (surgeons) preference for the referral of patients with CRLM to HPB surgeons and medical oncologists is problematic because the intricacies of CRLM management may not be practiced by all medical oncologists or HPB surgeons. In fact, previous research shows significant inconsistency among HPB surgeons with respect to management decisions for patients with CRLM [18]. The inconsistency surrounding management strategies and decisions of resectability is pronounced among non-HPB surgeons [19]. 

A recent consensus guideline was published by the International Hepato-Pancreato-Biliary Association (IHPBA), identifying that patients with non-resectable CRLM without extrahepatic metastatic disease or high-risk molecular criteria (BRAF V600E mutation, microsatellite stable, and mismatch repair proficient) who have shown a response to either 3 months of chemotherapy or 6 months of bridging therapy may be eligible for LT [20]. The importance of appropriate patient selection was also emphasized in these guidelines [20]. To improve consistency in referral patterns, evaluations of resectability, and management decisions for patients with CRLM, ongoing education, discussions, and development of consensus guidelines should be strived for both at the local and societal level [19]. 

Growing evidence shows that LT for non-resectable CRLM is surpassing the post-transplant 5-year OS of 50%, which is the threshold set for justifying the ethical use of LT for end-stage liver diseases [21,22]. The outcomes of LT for non-resectable CRLM only continue to improve with better patient selection based on biology, which was a topic of concern expressed by respondents to our survey [20]. For instance, the SECA-II trial conducted by the Oslo University Hospital Group used a more strict selection criteria (F-FDG PET/CT scan to evaluate metastases or local recurrence, at least 1-year from CRC diagnosis to LT listing, and at least 10% response on chemotherapy) for transplanting CRLM patients, and showed a promising 83% 5-year OS and 35% 5-year disease-free survival [23]. However, it should be noted that Norway has the distinct advantage of having a surplus of donor livers, which may make transplanting livers to CRLM patients more feasible [6]. In comparison, Canada is challenged with 21.8 deceased organ donors per million of the population, with 610 livers transplanted in 2019, but with a nearly 20% waiting list mortality [24].

To mitigate the effects of our national organ shortage and the metastatic nature of CRLM, our group at UHN is conducting a single-arm clinical trial assessing the combination of neoadjuvant chemotherapy and LDLT for non-resectable CRLM [10]. Despite the demands for more level I evidence observed in this survey, accrual to the LDLT CRLM trial remains a challenge due to stringent inclusion criteria. At UHN, all patients with CRLM referred are first assessed for resectability, including complex resections using Associating Liver Partition and Portal Vein Ligation for Staged Hepatectomy (ALPPS) following the IHPBA consensus guideline [20]. A recent editorial from our senior author (GS) highlights how the ALPPS maybe ideal for: (1) patients whose colon cancer remains in situ, and who need clearance of the left lobe; (2) patients with liver damage due to chemotherapy with a borderline future liver remnant; and (3) patients with a failed portal vein embolization [25]. Although an individual surgeon’s ability and preference might influence the treatment decision (ALPPS vs. LT), in the future, if LT becomes an accepted treatment for CRLM, we believe LT should only be offered to patients who are not eligible for any complex resection strategies. Our center previously demonstrated that LDLT provides long-term benefits, with similar graft survival rates as deceased donor LT [26]. Although the use of living donor livers may be an attractive solution to reduce waitlist mortality in countries such as Canada, research shows how center procedure volume correlates to complication rate, suggesting living donor liver surgeries should be limited to high volume centers [27]. 

Results from our study highlight a generally limited understanding and awareness of LDLT for non-resectable CRLM, and how the local HPB surgeon’s opinion may influence the CRC care provider’s final management or referral decisions. This identifies an opportunity to raise awareness of LDLT for non-resectable CRLM in several ways. First, succinct educational materials could be directly disseminated to general surgeons providing care for CRC patients in Canada to avoid CRLM patients being referred straight to palliative care without proper assessment by the HPB surgeon. We deemed that such an effort would be welcomed, based on 81% being interested in an education session on LDLT for CRLM, and could make a great impact, since nearly half of the respondents reported that they “might or might not consider” referral for LDLT for non-resectable CRLM patients. Second, the existing network of Canadian HPB surgeons could be utilized to further inform their local multidisciplinary cancer care teams to improve awareness of ongoing trials and initiatives for non-resectable CRLM to improve referral rates and comprehensive assessments. Future educational initiatives on this topic should also incorporate the medical oncology communities with multidisciplinary consensus on the management of non-resectable CRLM. In addition, future qualitative interview studies could investigate the attitudes of a select number of CRC care providers (colorectal and HPB surgeons, as well as medical oncologists) in greater depth than what was possible using a closed-ended study such as this.

The results from this study should be interpreted considering the following limitations. We were not able to calculate the accurate survey response rate as not every surgeon included in the mailing lists were CRC care providers that we were seeking, and there is significant overlap of members amongst the three societies we used (CAGS/CSCRS/CSSO). Furthermore, the survey invitation was indirectly delivered through mass email (CSSO) or mass newsletters (CAGS/CSCRS), in which the eligible members may not have read the invitation to participate in this study, potentially explaining the low numbers of total responses. However, our total number of responses was comparable to another national CRLM survey study that had used CAGS and CSCRS societies for survey delivery to surgeons (response *n* = 58) [28]. When interpretating our thematic extraction results, the response bias cannot not be eliminated, as this study may have attracted respondents who have stronger interest in this topic than others. In fact, 92% of our respondents identified themselves as surgical oncologists. Thus, the thematic extraction needs to be interpreted with caution, although a good mix of opinions were collected. Overall, we chose these three largest Canadian societies to broadly cover the nation, and make the results as generalizable as possible. In addition, the age category and number of years in training of respondents may suggest that some respondents are trainees (residents or fellows). We did not collect level of training, nor did we list any level of training as an inclusion or exclusion criterion, though the language of the survey and invitation suggested that our intended target audience was surgeons who had completed their clinical training. We cannot rule out the possibility that our results are skewed by trainee respondents.

## 5. Conclusions

In conclusion, this study partially identified a gap in knowledge and awareness of LDLT for non-resectable CRLM amongst CRC care providers across Canada. Informing CRC care providers about ongoing CRLM clinical trials and the most up-to-date evidence with educational materials on CRLM management may help raise the awareness of the LT option for non-resectable CRLM, and increase referral rates. Future studies should involve more granular assessments of surgeons’ opinions on this topic, using well-designed qualitative studies with structured interviews leading to theme saturations.

## Figures and Tables

**Table 1:** Demographic characteristics of responses.

Demographic Factor	*n* (%)
Total responses	37 (100)
Age (years)	
31–40	15 (40.5)
41–50	16 (43.2)
51–60	1 (2.7)
>60	2 (5.4)
Prefer not to say	3 (8.1)
Sex	
Male	22 (59.5)
Female	12 (32.4)
Non-binary	1 (2.7)
Prefer not to say	2 (5.4)
Years in practice	
<5	10 (27)
5–10	12 (32.4)
11–20	9 (24.3)
>20	6 (16.2)
Main practice specialty	
General Surgery	1 (2.7)
Surgical Oncology	34 (91.9)
Colorectal Surgery	2 (5.4)
Clinical practice setting	
Academic	28 (75.7)
Tertiary, non-academic	4 (10.8)
Community	5 (13.5)
Proportion of patients with CRLM	
0–25%	31 (83.8)
26–50%	4 (10.8)
51–75%	0 (0)
76–100%	2 (5.4)

CRLM = colorectal liver metastases.

**Table 2:** Current management strategies for patients with colorectal liver metastases.

Management Strategy	*n* (%)
Referral to medical oncologist for consideration for chemotherapy	27 (73)
Referral to hepatobiliary surgeon for consideration for metastatectomy	29 (78.4)
Referral to tertiary care surgical oncologist or colorectal surgeon	1 (2.7)
Presentation at multidisciplinary tumour rounds	25 (67.6)
Referral for other interventional therapy (e.g., portal vein embolization, tumour ablation, hepatic artery infusion pump chemotherapy)	3 (8.1)
Referral to palliative care	1 (2.7)
Other	3 (8.1)

Totals add to >100% because management strategy categories are not mutually exclusive.

**Table 3:** Knowledge, opinions, and attitudes towards living donor liver transplant for colorectal liver metastases.

Knowledge, Opinions, and Attitudes	*n* (%)
Self-rated knowledge about LDLT for CRLM	
Nothing	3 (8.1)
A little bit	18 (48.7)
Somewhat knowledgeable	13 (35.1)
Much	2 (5.4)
A great deal	1 (2.7)
Awareness of the indication for LDLT for CRLM	
Indications known	17 (45.9)
Indications not known	15 (40.5)
Unsure of indications	4 (10.8)
Perceived appropriateness of LDLT for CRLM	
Absolutely inappropriate	2 (5.4)
Inappropriate	8 (21.6)
Neutral	5 (13.5)
Appropriate	18 (48.7)
Absolutely appropriate	3 (8.1)
No response	1 (2.7)
Inclination to refer patients with CRLM, who are not candidates for metastectomy, for LDLT	
Would not consider	2 (5.4)
Might or might not consider	18 (48.7)
Would definitely consider	17 (45.9)
Surgeons interested in an education session on LDLT for CRLM	30 (81)

CRLM = colorectal liver metastases; LDLT = living donor liver transplant.

**Table 4:** Themes extracted from opinions related to perceived appropriateness of LDLT for CRLM.

Perceived Appropriateness of LDLT for CRLM	Themes
Inappropriate, or absolutely inappropriate	- Disease too advanced or tumor biology too poor for transplant to be effective - Potential benefit does not justify the risk or cost (i.e., to donor, patient, health care system) - Insufficient or negative data - Existence of better alternative therapies
Neutral	- Insufficient or negative data - Existence of better alternative therapies
Appropriate, or absolutely appropriate	- Appropriate only in context of clinical trial; insufficient data otherwise - Patients must be highly selected - Uncertain long-term outcomes

CRLM = colorectal liver metastases; LDLT = living donor liver transplant.

**Table 5:** Themes extracted from opinions related to surgeons’ inclination to refer patients for consideration for LDLT for CRLM.

Openness to LDLT for CRLM	Themes
Would not consider	- Existence of better alternative therapies - Desire for more data
Might or might not consider	- Desire for more data - Would consult colleague HPB physicians - Would only consider in context of clinical trial - Would refer if patient factors favorable
Would definitely consider	- Would consult colleague HPB physicians - Would only consider in context of clinical trial - Would refer if patient factors favorable

CRLM = colorectal liver metastases; HPB = hepatopancreatobiliary; LDLT = living donor liver transplant.

## Data Availability

Data are available directly from the authors upon reasonable request.

## Appendix A. Questionnaire

*Demographics*

1. What is your age (years)?
  - 20–30
  - 31–40
  - 41–50
  - 51–60
  - >60
  - Prefer not to say
2. What is your sex?
  - Male
  - Female
  - Non-binary
  - Other [TEXT BOX]
  - Prefer not to say
3. How many years have you been in practice?
  - <5 years
  - 5–10 years
  - 11–20 years
  - >20 years
4. What is your main practice specialty?
  - Surgical oncology
  - Colorectal surgery
  - General Surgery
  - Medical oncology
  - Other [TEXT BOX]
5. What proportion of your patients with colorectal cancer have colorectal liver metastases?
  - 0–25%
  - 26–50%
  - 51–75%
  - 76–100%
6. What is your clinical setting?
  - Academic
  - Tertiary, non-academic
  - Community

*Practice Patterns*

7. When you are the “Most Responsible Physician”, how do you manage a patient in whom you diagnose colorectal metastases confined to the liver (i.e., one or more metastatic deposits of colorectal cancer in the liver, but no distant metastases elsewhere in the body)? Choose all that apply.
  - Referral for management with chemotherapy
  - Referral to hepatobiliary surgery for consideration for metastectomy
  - Referral to a tertiary care surgical oncologist or colorectal surgeon
  - Presentation at multidisciplinary tumor rounds
  - Referral for other interventional therapy (e.g., portal vein embolization, tumor ablation, HAIP or Hepatic Arterial Infusion Pump Chemotherapy)
  - Referral to palliative care
  - Other [TEXT BOX]

*Opinions*

8. How much do you know about living donor liver transplant for potential curative management of isolated (confined to the liver) colorectal liver metastases? (Likert scale)
  - Nothing at all
  - A little bit
  - Somewhat
  - Much
  - A great deal
9. Are you aware of the indications for living donor liver transplant for isolated (confined to the liver) colorectal liver metastases?
  - Yes
  - No
  - Unsure
10. How appropriate do you think it is to treat isolated (confined to the liver) colorectal liver metastases with living donor liver transplant when metastectomy is contraindicated?
  - Absolutely inappropriate
  - Inappropriate
  - Neutral
  - Appropriate
  - Absolutely appropriate

COMMENT BOX—Why or Why not?

11. Would you consider referring a patient with isolated (confined to the liver) colorectal liver metastases, who is not a candidate for metastectomy, for consideration for living donor liver transplant (curative intent) if such a service were available? (Likert scale)
  - Would not consider
  - Might or might not consider
  - Would definitely consider

COMMENT BOX—Why or Why not?

12. Would you find an educational session helpful to promote awareness of LDLT for the treatment of colorectal liver metastases?
  - Yes
  - No

## References

[1] Rawla P., Sunkara T., Barsouk A. (2019). Epidemiology of Colorectal Cancer: Incidence, Mortality, Survival, and Risk Factors. Prz. Gastroenterol..

[2] Van der Geest L.G.M., Lam-Boer J., Koopman M., Verhoef C., Elferink M.A.G., de Wilt J.H.W. (2015). Nationwide Trends in Incidence, Treatment and Survival of Colorectal Cancer Patients with Synchronous Metastases. Clin. Exp. Metastasis.

[3] Riihimaki M., Hemminki A., Sundquist J., Hemminki K. (2016). Patterns of Metastasis in Colon and Rectal Cancer. Sci. Rep..

[4] Manfredi S., Lepage C., Hatem C., Coatmeur O., Faivre J., Bouvier A.M. (2006). Epidemiology and Management of Liver Metastases from Colorectal Cancer. Ann. Surg..

[5] Line P.D., Hagness M., Dueland S. (2018). The Potential Role of Liver Transplantation as a Treatment Option in Colorectal Liver Metastases. Can. J. Gastroenterol. Hepatol..

[6] Hagness M., Foss A., Line P.D., Scholz T., Jørgensen P.F., Fosby B., Boberg K.M., Mathisen Ø., Gladhaug I.P., Egge T.S. (2013). Liver Transplantation for Nonresectable Liver Metastases from Colorectal Cancer. Ann. Surg..

[7] Dueland S., Foss A., Solheim J.M., Hagness M., Line P.D. (2018). Survival Following Liver Transplantation for Liver-Only Colorectal Metastases Compared with Hepatocellular Carcinoma. J. Br. Surg..

[8] Sapisochin G., Hibi T., Ghobrial M., Man K. (2020). The ILTS Consensus Conference on Transplant Oncology: Setting the Stage. Transplantation.

[9] Giannis D., Sideris G., Kakos C.D., Katsaros I., Ziogas I.A. (2020). The Role of Liver Transplantation for Colorectal Liver Metastases: A Systematic Review and Pooled Analysis. Transplant. Rev..

[10] Sapisochin G. Assessment of a Protocol Using a Combination of Neo-Adjuvant Chemotherapy plus Living Donor Liver Transplantation for Non-Resectable Liver Metastases from Colorectal Cancer. https//clinicaltrials.gov/ct2/show/NCT02864485.

[11] Gorgen A., Muaddi H., Zhang W., McGilvray I., Gallinger S., Sapisochin G. (2018). The New Era of Transplant Oncology: Liver Transplantation for Nonresectable Colorectal Cancer Liver Metastases. Can. J. Gastroenterol. Hepatol..

[12] Berwick D.M. (2003). Disseminating Innovations in Health Care. J. Am. Med. Assoc..

[13] Lanari J., Dueland S., Line P.D. (2020). Liver Transplantation for Colorectal Liver Metastasis. Curr. Transplant. Rep..

[14] Harris P.A., Taylor R., Thielke R., Payne J., Gonzalez N., Conde J.G. (2009). Research Electronic Data Capture (REDCap)-A Metadata-Driven Methodology and Workflow Process for Providing Translational Research Informatics Support. J. Biomed. Inform..

[15] Abdelrahim M., Esmail A., Abudayyeh A., Murakami N., Saharia A., McMillan R., Victor D., Kodali S., Shetty A., Fong J.V.N. (2021). Transplant Oncology: An Evolving Field in Cancer Care. Cancers.

[16] Brenner D.R., Heer E., Sutherland R.L., Ruan Y., Tinmouth J., Heitman S.J., Hilsden R.J. (2019). National Trends in Colorectal Cancer Incidence among Older and Younger Adults in Canada. JAMA Netw. Open.

[17] Yong J.H.E., Mainprize J.G., Yaffe M.J., Ruan Y., Poirier A.E., Coldman A., Nadeau C., Iragorri N., Hilsden R.J., Brenner D.R. (2020). The Impact of Episodic Screening Interruption: COVID-19 and Population-Based Cancer Screening in Canada. J. Med. Screen..

[18] Ignatavicius P., Oberkofler C.E., Chapman W.C., DeMatteo R.P., Clary B.M., D’Angelica M.I., Tanabe K.K., Hong J.C., Aloia T.A., Pawlik T.M. (2020). Choices of Therapeutic Strategies for Colorectal Liver Metastases Among Expert Liver Surgeons: A Throw of the Dice?. Ann. Surg..

[19] Jones R.P., Vauthey J.-N., Adam R., Rees M., Berry D., Jackson R., Grimes N., Fenwick S.W., Poston G.J., Malik H.Z. (2012). Effect of Specialist Decision-Making on Treatment Strategies for Colorectal Liver Metastases. Br. J. Surg..

[20] Bonney G.K., Chew C.A., Lodge P., Hubbard J., Halazun K.J., Trunecka P., Muiesan P., Mirza D.F., Isaac J., Laing R.W. (2021). Liver Transplantation for Non-Resectable Colorectal Liver Metastases: The International Hepato-Pancreato-Biliary Association Consensus Guidelines. Lancet Gastroenterol. Hepatol..

[21] Schiano T.D., Bourgoise T., Rhodes R. (2015). High-Risk Liver Transplant Candidates: An Ethical Proposal on Where to Draw the Line. Liver Transplant..

[22] Moris D., Tsilimigras D.I., Chakedis J., Beal E.W., Felekouras E., Vernadakis S., Schizas D., Fung J.J., Pawlik T.M. (2017). Liver Transplantation for Unresectable Colorectal Liver Metastases: A Systematic Review. J. Surg. Oncol..

[23] Dueland S., Syversveen T., Solheim J.M., Solberg S., Grut H., Bjørnbeth B.A., Hagness M., Line P.D. (2020). Survival Following Liver Transplantation for Patients with Nonresectable Liver-Only Colorectal Metastases. Ann. Surg..

[24] Canadian Institute for Health Information (2020). Annual Statistics on Organ Replacement in Canada: Dialysis, Transplantation and Donation, 2010 to 2019.

[25] Sapisochin G. (2021). To Associating Liver Partition and Portal Vein Ligation for Staged Hepatectomy (ALPPS) or Not to ALPPS. Surgery.

[26] Goto T., Ivanics T., Cattral M.S., Reichman T., Ghanekar A., Sapisochin G., McGilvray I.D., Sayed B., Lilly L., Bhat M. (2021). Superior Long-Term Outcome of Adult Living Donor Liver Transplantation A Cumulative Single-Center Cohort Study with 20 Years Follow-Up. Liver Transpl..

[27] Rössler F., Sapisochin G., Song G.W., Lin Y.H., Simpson M.A., Hasegawa K., Laurenzi A., Cabús S.S., Nunez M.I., Gatti A. (2016). Defining Benchmarks for Major Liver Surgery: A Multicenter Analysis of 5202 Living Liver Donors. Ann. Surg..

[28] Howard C., Clements T.W., Edwards J.P., MacLean A.R., Buie W.D., Dixon E., Grondin S.C., Gomes A., McColl M., Cleary S.P. (2018). Synchronous Colorectal Liver Metastases: A National Survey of Surgeon Opinions on Simultaneous Resection and Multidisciplinary Cooperation. Hepatobiliary Surg. Nutr..

